# Using Molecular Diagnostics to Develop Therapeutic Strategies for Carbapenem-Resistant Gram-Negative Infections

**DOI:** 10.3389/fcimb.2021.715821

**Published:** 2021-09-28

**Authors:** Fred C. Tenover

**Affiliations:** Cepheid, Sunnyvale, CA, United States

**Keywords:** carbapenems, carbapenemase, susceptibility testing, syndromic panels, ESBL, AmpC, mCIM

## Abstract

Infections caused by multidrug-resistant Gram-negative organisms have become a global threat. Such infections can be very difficult to treat, especially when they are caused by carbapenemase-producing organisms (CPO). Since infections caused by CPO tend to have worse outcomes than non-CPO infections, it is important to identify the type of carbapenemase present in the isolate or at least the Ambler Class (i.e., A, B, or D), to optimize therapy. Many of the newer beta-lactam/beta-lactamase inhibitor combinations are not active against organisms carrying Class B metallo-enzymes, so differentiating organisms with Class A or D carbapenemases from those with Class B enzymes rapidly is critical. Using molecular tests to detect and differentiate carbapenem-resistance genes (CRG) in bacterial isolates provides fast and actionable results, but utilization of these tests globally appears to be low. Detecting CRG directly in positive blood culture bottles or in syndromic panels coupled with bacterial identification are helpful when results are positive, however, even negative results can provide guidance for anti-infective therapy for key organism-drug combinations when linked to local epidemiology. This perspective will focus on the reluctance of laboratories to use molecular tests as aids to developing therapeutic strategies for infections caused by carbapenem-resistant organisms and how to overcome that reluctance.

## Introduction

Infections caused by multidrug-resistant Gram-negative organisms have become a global threat (Logan and Weinstein, 2017). Such infections can be very difficult to treat, especially when they are caused by carbapenemase-producing organisms (CPO) (Tamma et al., 2021). Outcomes of patients with infections caused by CPO are worse than those caused by bacteria that are resistant to carbapenems by other mechanisms, such as efflux or permeability changes in the cell’s outer membrane. For example, Tamma et al. reported that the odds of a patient dying from bacteremia within 14 days of disease onset was 4 times greater with a CPO infection than with a non-CPO infection (Tamma et al., 2017). Thus, it is important to differentiate between CPO and non-CPO infections to help guide treatment of individual patients and improve outcomes, especially for sepsis. While detection of CPO can be accomplished with phenotypic or genotypic methods, this perspective will focus on molecular genotypic methods.

Molecular diagnostic tests can inform therapeutic decisions for bacterial infections in two ways. First, colonies of carbapenem-resistant gram-negative organisms recovered from clinical specimens in pure culture can be tested to confirm the presence of a carbapenem-resistance gene (CRG) and to differentiate between serine carbapenemase genes and metallo-carbapenemase genes (Lutgring and Limbago, 2016; Iovleva and Doi, 2017; Traczewski et al., 2018). These are critical data for clinicians as many of the newer beta-lactam/beta-lactamase inhibitor combinations are not active against metallo-beta-lactamase producing bacteria. The second application of molecular diagnostics is to couple detection of CRGs with bacterial species identification in syndromic blood culture and respiratory panels to guide therapy. Both applications will be explored in this perspective.

## Differentiating Among Ambler Classes of Carbapenem-Resistance Genes

When treating an infection caused by a CPO, differentiating between resistance caused by Class A enzymes, such as the *Klebsiella pneumoniae* carbapenemase (KPC) and Class D enzymes, such as Oxacillinase-48 (OXA-48), *versus* Class B enzymes, including the New Delhi metallo-beta-lactamase (NDM), Verona Integron-encoded metallo-beta-lactamase (VIM), and Imipenemases (IMP) is critical especially when physicians consider using any of the newer beta-lactam/beta-lactamase inhibitor combinations (Falcone and Paterson, 2016; Sheu et al., 2019; Ackley et al., 2020; Johnston et al., 2020). Many of the newer combination agents, including ceftazidime-avibactam, ceftolozane-tazobactam, meropenem-vaborbactam, and imipenem-cilastatin-relebactam, are not active against the Class B metallo-carbapenemases (**Table 1**). Thus, optimal use of these new and expensive anti-infective agents requires knowledge of the mechanism of carbapenem resistance in the target organism. Yet, published data suggest that differentiation among beta-lactamase classes in CPOs often is not performed (Lutgring and Limbago, 2016; Miller et al., 2017; Burnham et al., 2017; Pereckaite et al., 2018). The underutilization of these tests is a critical problem for optimal use of the newer antimicrobial agents globally.

**Table 1:** Activity of recent beta-lactam/beta-lactamase inhibitor combinations against microorganisms containing carbapenemases^a^.

Antimicrobial Agent	FDA status^a^	EMA status^b^	Carbapenemase (Class)				
			KPC (A)	NDM (B)	IMP (B)	VIM (B)	OXA-48 (D)
Ceftzidime-avibactam	Approved	Authorized	Yes	No	No	No	Limited
Meropenem-vaborbactam	Approved	Authorized	Yes	No	No	No	No
Ceftolozane-tazobactam	Approved	Authorized	No	No	No	No	No
Imipenem-cilastatin-relebactam	Approved	Authorized	Yes	No	No	No	No
Cefiderocol	Approved	Authorized	Yes	Yes	Yes	Yes	Yes
Aztreonam-avibactam	Phase III clinical trial	Authorized	Yes	Yes	Yes	Yes	Yes

a Adapted from https://www.centerwatch.com/directories/1067-fda-approved-drugs/topic/116-infections-and-infectious-diseases accessed 4-8-2021.

b European Medicines Agency; https://www.ema.europa.eu/en/medicines/human accessed 6-19-2021.

## Reluctance to Using Molecular Diagnostic Tests for CPO Detection

There are several reasons why laboratories may be reluctant to use molecular tests to detect CPOs and differentiate among CRG classes in resistant bacterial isolates. The cost of the tests is often cited as an issue, especially for the genotypic assays (Burnham et al., 2017; Pereckaite et al., 2018) but the reluctance to use molecular tests clearly goes beyond this issue as syndromic test panels are broadly used in clinical microbiology laboratories despite their increased costs over traditional identification methods (Dien Bard and McElvania, 2020). For many laboratories in the United States, there is a sense that differentiation among CRG is simply unnecessary, as it is presumed that CPOs are likely to be KPC-producers (Miller et al., 2017). Unfortunately, this is not necessarily true as the epidemiology of KPC-producing organisms is very uneven in the U.S (Lutgring et al., 2018) and other carbapenemases, including VIM, NDM, IMP, and OXA-48, have been detected in multiple U.S. states and their prevalence has steadily increased (Logan and Bonomo, 2016; Lutgring and Limbago, 2016; Logan and Weinstein, 2017). Furthermore, Miller et al. reported that many laboratories assume that any gram-negative Enterobacteriaceae isolate that tests as resistant to any carbapenem is a carbapenemases producer (CPE). This is based partially on the Centers for Disease Control and Prevention (CDC) definitions for CPE (Miller et al., 2017). However, detection of carbapenemase genes or carbapenemase activity in isolates is also part of the CDC’s CPE definition, but this is often overlooked. Thus, the terms CRE (a broad category) and CPE (only carbapenemase producers) are often confused. While the sensitivity of the CDC definition for CPE based on susceptibility testing alone was 100% in this study, the specificity was only 6.1%, which means CRG-mediated resistance was dramatically over reported, which negatively impacts therapeutic choices (Tamma et al., 2021). According to Miller et al., the specificity of testing could be raised to ~100% by using either a phenotypic test, such as the modified carbapenem inactivation method (mCIM) to detect carbapenemase activity in colonies (without beta-lactamase class differentiation), or a commercial genotypic test, such as the Xpert^®^ Carba-R test (Cepheid, Sunnyvale, CA, USA)(IVD – *in vitro* diagnostic test] (Traczewski et al., 2018) to detect and differentiate among the genes encoding five major classes of carbapenemases. The high specificity of testing is needed to prevent the misuse of the novel beta-lactam/beta-lactamase inhibitor combinations. Data on the use of molecular methods to detect CPO in the European Union other parts of the world are scarce (Pereckaite et al., 2018).

A second source of reluctance to using molecular tests is the presumption that carbapenem resistance in isolates of *Pseudomonas aeruginosa* and *Acinetobacter* species is not mediated by CRG (Gill et al., 2021). Although both *Pseudomonas* and *Acinetobacter* species have permeability- and efflux-mediated carbapenem resistance, they also can harbor a wealth of CRG, some of which are difficult to detect with phenotypic methods (Jahan et al., 2020; Sharma et al., 2020). Those genes negatively impact the use of beta-lactam/beta-lactamase inhibitor combinations, since most of the enzymes reported in these species are Class B metallo-beta-lactamases (Wang et al., 2021). The widespread use of ceftolozane/tazobactam for *P. aeruginosa* infections is particularly worrisome since this agent is not active against CPO of any class (**Table 1**) (Jorgensen et al., 2020).

Reluctance to using molecular tests also comes from the confusion over which tests have received regulatory approval for developing therapeutic strategies for infections *versus* tests approved only to guide infection control interventions. Most tests to detect carbapenemases or CRG have only received regulatory clearance to guide infection control activities and not for guiding therapeutic decisions. Using tests only for the purposes described in the product’s instructions for use (IFU) is an important factor for many laboratories in an increasingly regulated laboratory environment. For example, in the United States, the NG-Test^®^ CARBA-5 (Hardy Diagnostics, Santa Maria, CA) IFU says it “…is intended as an aid for infection control in the detection of carbapenamase-producing *Enterobacteriaceae* and *Pseudomonas aeruginosa* in healthcare settings. [It] is not intended to guide or monitor treatment for carbapenem-non-susceptible bacterial infections” (Hardy Diagnsotics, 2019). In contrast, the Xpert^®^ Carba-R test has received US-IVD clearance for use as an aid for infection control and “… for guiding therapeutic strategies, but only when testing carbapenem-non-susceptible colonies from pure cultures” (Cepheid, 2019). As noted in the recent Italian guidelines for CRE prevention, it is important for laboratories to be familiar with the intended use statement of all commercial tests to make sure they are using them appropriately (Ambretti et al., 2019). Use of an IVD outside of its intended use requires a validation study (Burd, 2010). Other products that have received regulatory clearance for developing therapeutic strategies for infections caused by CPO are available in the European Union and elsewhere.

## Using Algorithms to Detect CPOs and Differentiate Among CRG in Pure Cultures of Bacteria

Laboratories do not always know which test is optimal for their needs and are concerned about the accuracy of a single test. Given the diversity of beta-lactamases circulating globally, laboratories may need to use a combination of phenotypic and genotypic tests to optimize detection of carbapenem resistance mechanisms. This is especially important when both serine- and metallo-beta-lactamase genes are present among clinical isolates in a region. Khalifa et al. (2020) described an algorithm using methods available in the European Union that started with a rapid commercial test to detect IMP, KPC, NDM, OXA-48, and VIM, such as the CARBA-5 lateral flow immunochromatographic assay, or a genotypic test, such as the Xpert^®^ Carba-R PCR test (**Table 2**). If the rapid test is negative, they recommend the mCIM test (Pierce et al., 2017) to determine if the organism is a CPO with a novel carbapenemase (Clinical and Laboratory Standards Institute, 2020). The algorithm offers rapid turn-around time (<1 hour for many of the commercial tests) for differentiation of most serine and metallo-carbapenemases coupled with the ability to detect novel carbapenemases with the mCIM test. This approach indicates those anti-infective agents that are not active against the metallo-beta-lactamases (**Table 1**) *versus* novel agents like cefideracol, a cephalosporin-siderophore combination with broad activity that includes many organisms that harbor either serine or metallo-enzymes (Hackel et al., 2018). Using the mCIM test together with the eCIM test in which 5mM ethylene-diamine tetra acetic acid (EDTA) is added to a second meropenem disk, can broadly differentiate a serine beta-lactamase from a metallo-beta-lactamase (Sfeir et al., 2019) but this approach doesn’t indicate which resistance gene is present. Nonetheless, this simple combination of disk tests is cost-effective and has a high degree of accuracy (Tenover et al., 2020).

**Table 2:** Examples of molecular and immunochromatographic diagnostic tests to detect and differentiate carbapenem resistance genes in pure culture colonies or clinical specimens.

Test name; Manufacturer	Technology; Specimen types; availability^a^	Carbapenem resistance genes detected
Xpert^®^ Carba-R; Cepheid, Sunnyvale, CA	NAAT; Pure cultures of carbapenem-resistant organisms, rectal swabs, peri-rectal swabs; EU and US	*bla*_IMP_, *bla*_KPC_, *bla*_NDM_, *bla*_OXA-48-like_, and *bla*_VIM_
CARBA-5; NG Biotech, Guipry, France	Immunochromatographic; Pure cultures of carbapenem-resistant organisms; EU and US	*bla*_IMP_, *bla*_KPC_, *bla*_NDM_, *bla*_OXA-48-like_, and *bla*_VIM_
BioFire BCID2; BioFire, Salt Lake City, UT, USA	Film array; Blood culture bottles; EU and US	*bla*_KPC_, *bla*_IMP_, *bla*_NDM_, *bla*_OXA-48-like_, and *bla*_VIM_
Luminex Verigene BC-GN; Luminex, Toronto, CA	NAAT; Blood culture bottles; EU and US	*bla*_KPC_, *bla*_IMP_, *bla*_NDM_, *bla*_OXA-48_, and *bla*_VIM_
GenMark ePlex BCID-GN; Carlsbad, CA, USA	NAAT; Blood culture bottles; EU and US	*bla*_KPC_, *bla*_IMP_, *bla*_NDM_ *bla*_OXA-23_, *bla*_OXA-48_, and *bla*_VIM_
iCubate iC-GN; iCubate, Huntsville, AL, USA	NAAT; Blood culture bottles; US	*bla*_KPC_ and *bla*_NDM_
BioFire Pneumonia panel (BioFire)	Film array; Respiratory specimens; EU and US	*bla*_IMP_, *bla*_KPC_, *bla*_NDM_, *bla*_OXA-48-like_, and *bla*_VIM,_
Unyvero LRT panel; Curetis, Gaithersburg, MD, USA	NAAT; Respiratory specimens EU and US	*bla*_KPC_, *bla*_NDM_, *bla*_OXA-23_, *bla*_OXA-24_, *bla*_OXA-48_, *bla*_OXA-58_, and *bla*_VIM_

a NAAT, nucleic acid amplification test; Data adapted from company websites.

## Molecular Tests to Identify Resistance Genes in Positive Blood Culture Bottles And Respiratory Specimens

Using the presence or absence of a resistance gene in a clinical specimen prior to the isolation of a bacterial isolate to predict phenotypic susceptibility or resistance to specific antimicrobial agents is more complicated than distinguishing between CPO and non-CPO. The dilemma of reconciling conflicting genotypic and phenotypic data has recently been reviewed by Yee et al. (2021). Detection of a CRG has a high positive predictive value as an indicator of phenotypic resistance in most bacterial species; however, the absence of a resistance gene does not always indicate that the organism detected will be phenotypically susceptible to an antibiotic. The problems are illustrated in an exchange between Spafford et al. (Spafford et al., 2019; Humphries et al., 2020) and Pogue and Heil (2020), regarding the use of genotypic tests for *bla*_CTX-M_, *bla*_KPC_, and *bla*_NDM_ in blood culture panels to predict phenotypic resistance or susceptibility to extended-spectrum cephalosporins and carbapenems in gram-negative organisms. The high very major error rates (which indicate false susceptibility to an antimicrobial agent) and variable negative predictive values of the molecular tests by geographic region and hospital type, led Spafford et al. to raise flags of caution regarding the interpretation of genotypic results. In response, Pogue and Heil defended the use of the genotypic tests for specific predetermined organism-drug combinations, which was supported by their studies in two different hospitals (Pogue et al., 2018), where, with the exception of the *P. aeruginosa* and pipericillin/tazobactam combination, the negative predictive values for genotypic data were quite high for selected resistance gene-organism-antimicrobial agent combinations. In other words, the absence of a resistance gene could predict susceptibility of a bacterial species to one or more antimicrobial agents, but the predictive value may differ from hospital to hospital depending on the prevalence of the resistance genes that are included in the syndromic panel.

Unfortunately, the data supporting the value of including CRG in syndromic panels (several of which are listed in **Table 2**) to improve anti-infective therapy currently remain sparse (Dien Bard and McElvania, 2020). In many reports, the low prevalence of CPO containing a CRG of interest has hindered the accumulation of outcome data. Thus, the continued study of the impacts of syndromic panels on improving the outcomes of patients should be focused on defining the resistance gene-species-antimicrobial agent combinations that yield high predictive values. This is an area that requires well-controlled trials, preferably in multiple geographic regions.

## Discussion

There are multiple phenotypic and molecular genotypic tests (Tamma and Simner, 2018) in addition to those listed above that can be used to optimize therapeutic regimens. This includes the use of matrix-assisted laser desorption/ionization time-of-flight (MALDI-TOF) mass spectrometry to identify microorganisms directly in positive blood culture bottles (Patel, 2015) and to identify proteins associated with antimicrobial resistance mechanisms, including carbapenemases, such as KPC-2, in a rapid and cost effective manner (Gaibani et al., 2016; Vrioni et al., 2018; Figueroa-Espinosa et al., 2019). Earlier differentiation between CPO and non-CPO enhances the ability of antimicrobial stewardship programs to move from empiric to directed antimicrobial therapy to treat infections. While molecular tests have been shown to have value, their limitations, such as cost, the need for new instrumentation, and limited spectrum of gene detection (only known resistance genes are detected) must be noted. Nevertheless, the limitations of the molecular tests can be offset to a certain degree by their high sensitivity and specificity, rapid turn-around-time, and the ability to guide therapy early in the course of infection (Burnham et al., 2017; Ambretti et al., 2019). Unfortunately, these tests receive only cursory mention in key guidelines, such as those from the Clinical and Laboratory Standards Institute, the European Committee on Antimicrobial Susceptibility Testing, the CDC, and the Infectious Diseases Society of America’s guideline for treating infections caused by multidrug resistant organisms (Tamma et al., 2021). Thus, the tests are underutilized. While these organizations cannot promote specific commercial tests, they should promote the concept of detecting CPOs as early as possible in the course of infection and differentiating among enzyme classes to ensure the prudent use of the newer beta-lactam/beta-lactamase inhibitor combinations and enhance antimicrobial stewardship efforts globally.

In summary, tests performed on bacterial colonies of carbapenem-resistant organisms can indicate whether the isolate is a CPO and if so, whether resistance is mediated by a Class A, B, or D carbapenemase. While the positive predictive value of identifying CRGs in parallel with bacterial species identification in syndromic panels is high, the negative predictive value, although lower, can also be very useful if one considers the prevalence of the resistance gene in specific organisms for specific antimicrobial agents. Better education programs for microbiologists, physicians, and pharmacists are needed to clarify the differences between CRO and CPO and how to optimize therapeutic strategies for infections caused by these two groups of organisms.

## Data Availability Statement

The original contributions presented in the study are included in the article/supplementary material. Further inquiries can be directed to the corresponding author.

## Author Contributions

The author confirms being the sole contributor of this work and has approved it for publication.

## Funding

Funding for the development of this article was provided by Cepheid. The funder was not involved in the study design, collection, analysis, interpretation of data, the writing of this article or the decision to submit it for publication.

## Conflict of Interest

Author FT was employed by Cepheid.

## Publisher’s Note

All claims expressed in this article are solely those of the authors and do not necessarily represent those of their affiliated organizations, or those of the publisher, the editors and the reviewers. Any product that may be evaluated in this article, or claim that may be made by its manufacturer, is not guaranteed or endorsed by the publisher.
